# Transience effect in capture-recapture studies: The importance of its biological meaning

**DOI:** 10.1371/journal.pone.0222241

**Published:** 2019-09-19

**Authors:** Meritxell Genovart, Roger Pradel

**Affiliations:** 1 CEAB (CSIC), Theoretical and Computational Ecology, Blanes, Catalonia, Spain; 2 IMEDEA (CSIC-UIB), Esporles, Spain; 3 CEFE, CNRS, University Montpellier, University Paul Valéry Montpellier 3, EPHE, IRD, Montpellier, France; Universitat Zurich, SWITZERLAND

## Abstract

Capture–recapture (CR) models are an essential tool for estimating demographic parameters in most animal and some plant populations. To avoid drawing incorrect conclusions in any statistical inference, a crucial prerequisite is to assess the goodness of fit of a general model to the data. In CR models, a frequent cause of lack of fit, is the so-called *transience effect*, which is due to a lower expectation of re-observation of individuals marked for the first time as compared to other individuals. The transience effect may result either from different biological causes or from the sampling procedure. A transience effect is usually treated by distinguishing at least two age-classes in the survival probability, but other approaches may be more suitable. Here we develop a conceptual and analytical framework for including a transience effect in capture-recapture models. We show the implementation of two additional parametrizations that incorporate a transience effect. With these parametrizations, we can directly estimate the “transience probability” defined as the probability that a newly caught individual disappear from the population beyond what is expected based on the behavior of the previously caught individuals in the same sample. Additionally, these parametrizations allow testing biological hypotheses concerning drivers affecting this probability. Results from our case study show differences between parametrizations, with the parametrization most currently used giving different estimates, especially when including covariates. We advocate for a unifying framework for treating a transience effect, that helps clarifying the ideas and terminology, and where the biological reasons should be the rule for choosing the appropriate analytical procedure. This framework will also open new and powerful ways for the detection and exploration of ecological processes such as the costs of the first reproduction or the deleterious effects of some types of marking.

## Introduction

Capture–recapture (CR) models are an essential tool for estimating and analyzing factors driving some demographic parameters (e.g. survival, recruitment, dispersal) in most animal populations and some plant populations [1,2]. As in any statistical inference, in CR models, assessing the goodness of fit (GOF) of a general model to the data is a crucial prerequisite to avoid drawing incorrect conclusions [3]. Significant lack of fit of the general model should force to investigate the reasons for this lack of fit and adapt the model accordingly. CR models are based on four key assumptions [1, 4], sometimes known as the ‘Cormack-Jolly-Seber assumptions’: 1) individual marks are not lost or missed; 2) all samples are instantaneous relative to the interval separating occasions, and each release is made immediately after the sample; 3) every marked animal present in the population at time (i) has the same probability of recapture; 4) every marked animal in the population immediately after time (i) has the same probability of surviving to time (i +1)[5,6]. For most capture-recapture studies we know or confidently accept that assumptions 1 and 2 are met, and assumptions 3 and 4 are typically the most important in terms of GOF testing (6). GOF tests are present as part of the available software programs for capture-recapture data goodness-of-fit testing or in specialized R packages [7,8]. A violation of one of these tests, TEST 3.SR, is the so called *transience effect*, meaning that the individuals captured for the first time (‘new individuals’) have a lower expectation of being re-observed in the future, as compared to individuals of the same sample that had been captured previously (‘old individuals’) [9]. As an example, in a review of CR studies analyzing data from breeding sites, Oro and Doak [10] found that a transience effect was detected 37% of the times. In most cases, this heterogeneity in local survival is treated by considering at least two age classes in the survival probability. However, this procedure may not always reflect adequately the underlying biological phenomenon. Even if very rarely used (but see [11–13]), there are other ways to account for a transience effect, which may be more appropriate depending on the biological phenomenon that caused transience, and we may want to approach the issue from different perspectives. With these other parametrizations, we could directly estimate the “transience probability” i.e. the probability that a newly caught individual disappear from the population (die or permanently emigrate) beyond what is expected based on the behavior of the previously caught individuals from the same sample. With these parametrizations we could even test biological hypotheses concerning the external drivers (e.g. climate) and internal factors (e.g. age, sex) affecting this probability. Additionally, terminology around transience is actually ambiguous (Box 1). We think that there is a need to distinguish the statistical transience effect from its potential biological causes, of which the presence of transients as individuals transiting through the area, i.e. non-resident individuals, is only one possibility. Here we expose different possible ways to incorporate a transience effect in capture-recapture models and different biological reasons that may be behind it, and suggest the best analytical way to include this effect in our models in each case. Our main goal is to provide an updated conceptual and analytical framework to deal with transience effects in CR studies.

**Box 1. Glossary**

**Goodness-of-fit test** of a statistical model (**GOF**): test to summarize the discrepancy between observed values and the values expected under the model in question.

**Test 3SR**: also called the Brownie–Robson test. Is a subcomponent of the CJS goodness–of–fit test, and also a specific test of transience in single-site/state models, that checks that newly and already–marked animals captured at the same time have an equal chance of being ever reobserved.

**Transients**: individuals never again available for recapture after initial capture. Operationally defined as individuals having a zero probability of local survival after their initial capture. They may be individuals transiting through but not belonging to the study population (true transients), individuals that only reproduce once and then die or permanently disperse (cost of first reproduction), or individuals that die or permanently disperse due to an effect of marking (marking effect).

**Transience effect**: A deviation on the TEST 3.SR, hence essentially a statistical concept. Individuals captured for the first time (‘new individuals’) have a lower expectation of being reobserved in the future as compared to individuals of the same sample that had been captured previously (‘old individuals’).

**Transience probability**: the probability that a newly caught individual disappears from the population (die or permanently emigrate) beyond what is expected based on the behavior of the previously caught individuals from the same sample. Depending on the context, this probability corresponds to the probability of transition at first capture to the state ‘transient’ for individuals initially identical (parametrization C in article), or the proportion of transients among the newly marked for a population made of a mixture of true transients and residents (parametrization B). In case parametrization A has been used, the probability to become a transient (C), or the proportion of transients among the newly marked (B) can be obtained as 1-(phi(1)/phi(2)), where phi(1) is the apparent survival of first captured individuals and phi(2) is the apparent survival of previously captured individuals.

## Conceptual and analytical framework

### Biological meanings of a transience effect

A deviation on the TEST 3.SR may result from different biological causes or even relate to the marking procedure. We enumerate below the main biological explanations for the presence of a transience effect in the data.

#### Age

In many species, survival or permanent dispersal may be different for individuals of different ages, thus if for example individuals are marked as young or juveniles, the lower probability of reencounter of newly captured individuals may be due to an age effect (i.e. juveniles have lower survival or higher permanent dispersal) (i.e.[14]). In this case, the terminology “residents and transients” is not adequate. In cases when we are not interested in estimating parameters of younger individuals, we may want to remove the first capture in all capture histories. This simplifies the model (no age effect), at the expense of some loss of information.

#### True transients

In some cases, some individuals included in our data set may not strictly belong to the study population; they are transiting through the study area. In this case, the terminology “residents and transients” is adequate, as some individuals only seen once are transients, whereas those seen on subsequent occasions are all residents [15]. This may occur at breeding sites (i.e. colonies for birds and mammals or ponds for amphibians) with some captured individuals being breeders (residents) and others just individuals caught transiting the area (transients) [12,16,17]; another example of the analysis of true transients would be some studies at stopover sites or wintering areas, where residents (i.e. those making a stopover or hibernating) and transients are often present [18]. Additionally, for example, when studying a population at its breeding place but not strictly marking individuals when found breeding (e.g. at the nest or burrow), some sampling methods (e.g. birdcalls or decoys) may increase the transience effect by enlarging the number of individuals attracted and not belonging to the study population (e.g.[19]). When true transients occur, we would like to estimate our demographic parameters of interest (e.g. survival) without taking into account information from these individuals. We may also be interested in estimating the proportion of real transients in the population, and in determining the factors affecting it.

#### Marking effect

Also marking an individual may affect it in such a way that it decreases survival or induces permanent emigration. This would be a researcher effect and can only occur when manipulation takes place for marking. For instance, it has been demonstrated that, following the helicopter roundup of moulting adults of lesser snow geese accompanied by non-flying goslings, 25 to 30% of the birds undergoing this likely stressful procedure for the first time emigrate permanently [20]. Apart from including this capture effect in our models to get reliable demographic parameter estimates, we may also be interested in estimating this transience probability, in this case the probability of emigration or additional mortality due to the marking effect, and in determining the factors affecting it, external and internal.

#### Cost of first reproduction

Reproduction is costly, especially first time reproduction, and the presence of a link between survival and reproduction is a concept underpinning the theory of life-history evolution [21,22]. When many animals enter the marked population as first-time breeders, a transience effect can indicate a cost of first reproduction. This is because individuals breeding for the first time may have a higher mortality or permanent dispersal probability than experienced breeders (e.g. [23]). We may be interested in estimating this transience probability in first-time breeders and exploring the factors affecting the costs of the first reproduction.

### Multievent parametrizations

Models dealing with transience can be implemented as Multievent models [24] or equivalently as state-space models [25,26]. In the following, we build our models in the Multievent framework, but the same conceptual ideas can be implemented as state space models. Multievent models hold two levels, 1) the field observations called “events” encoded in the capture histories, and 2) the “states” defined to match the biological questions; those can only be inferred.

We describe three general parametrizations that include a transience effect in our models. The first one is the most vastly used approach, and involves including at least two age classes when analyzing survival probabilities (“Transience effect as an age effect”; parametrization A). The second one treats transience as a different individual state, thus, there would be individuals “transients” and others “residents” (“Transience effect as a state”; parametrization B). In this parametrization, individuals are intrinsically transients or residents before and independently of the first observation. The third analytical procedure analyzes transience as a possible transition for those individuals captured for the first time (“Transience effect as a transition”; parametrization C). In this parametrization, the change of state occurs after this first sampling occasion and may result from the sampling procedure itself (i.e. effect of marking) or from other experiences undergone simultaneously to this first sampling occasion (e.g. effect of first breeding attempt). We describe below the implementation of the three mentioned Multievent parametrizations to account for a transience effect. For all models, the symbols for parameters are: ϕ: survival probability, *Tr*: Transience probability, which is context dependent, *p*: Recapture probability. Several kinds of dependency may be considered on these parameters (e.g. constancy, time dependency, or dependence on individual or environmental covariates). Models are described by means of row-stochastic matrices, i.e. each row contains the parameters of a multinomial distribution. Consequently, the total of cell probabilities per row is 1. Because of this constraint, one and only one cell probability in each row will be calculated as the complement to 1 of the others. For the sake of clarity, all models are kept as simple as possible; we only correct for a transience effect, we do not consider groups, and, except for parametrization A, we do not consider age effects. But these models can be modified to correct for a trap effect if the GOF testing points to such an effect [27], and include as much complexity as needed, in terms of states and observations, to answer our research question.

#### Transience as an age effect (A)

The individual states considered are: Individual alive (A) and dead (D), this last state not being observable. The possible events are: not seen (0) and seen (1). Even if not essential from a mathematical point of view, from a conceptual point of view, to include a transience effect as an age effect, individuals captured for the first time should be younger that those seen on later occasions (i.e. individuals marked as juveniles). In this parametrization, the initial state, i.e. the state at first encounter of an individual, may only be A.

Initial state:

$$\begin{array}{cc} A & D\\ 1 & 0 \end{array}$$

We have one transition matrix, which models survival probabilities from the state at *t* (in row) to the state at *t*+1 (in column):

AD

$$S=\begin{array}{c} A\\ D \end{array}\left(\begin{array}{cc} \emptyset & 1-\emptyset \\ 0 & 1 \end{array}\right)$$

And the matrix of event probabilities (E) relating states in row and events in columns:

01

$$E=\begin{array}{c} A\\ D \end{array}\left(\begin{array}{cc} 1-p & p\\ 1 & 0 \end{array}\right)$$

In this parametrization, the transience effect is rendered by considering at least two age classes.

#### Transience as a state (B)

In this parametrization, transience is seen as a preexisting individual state. The individual states considered are: Individual transient (AT), individual resident (AR) and dead (D), this last state not being observable. In all parametrizations, but especially in this case, the dead state (D) does not necessarily imply the death of the individual but more generally represents the permanent departure of the individual from the study population. The possible events are: not seen (0) and seen (1). In this parametrization, the initial state, i.e. the state at first encounter of an individual, may be AT or AR.

Initial State:

$$\begin{array}{ccc} AT & AR & D\\ Tr & 1-Tr & 0 \end{array}$$

Transition matrix:

ATARD

$$S=\begin{array}{c} AT\\ AR\\ D \end{array}\left(\begin{array}{ccc} 0 & 0 & 1\\ 0 & \emptyset & 1-\\ 0 & 0 & 1 \end{array}\emptyset \right)$$

Event probabilities matrix:

01

$$E=\begin{array}{c} AT\\ AR\\ D \end{array}\left(\begin{array}{cc} 1-p & p\\ 1-p & p\\ 1 & 0 \end{array}\right)$$

**Fig 1:**
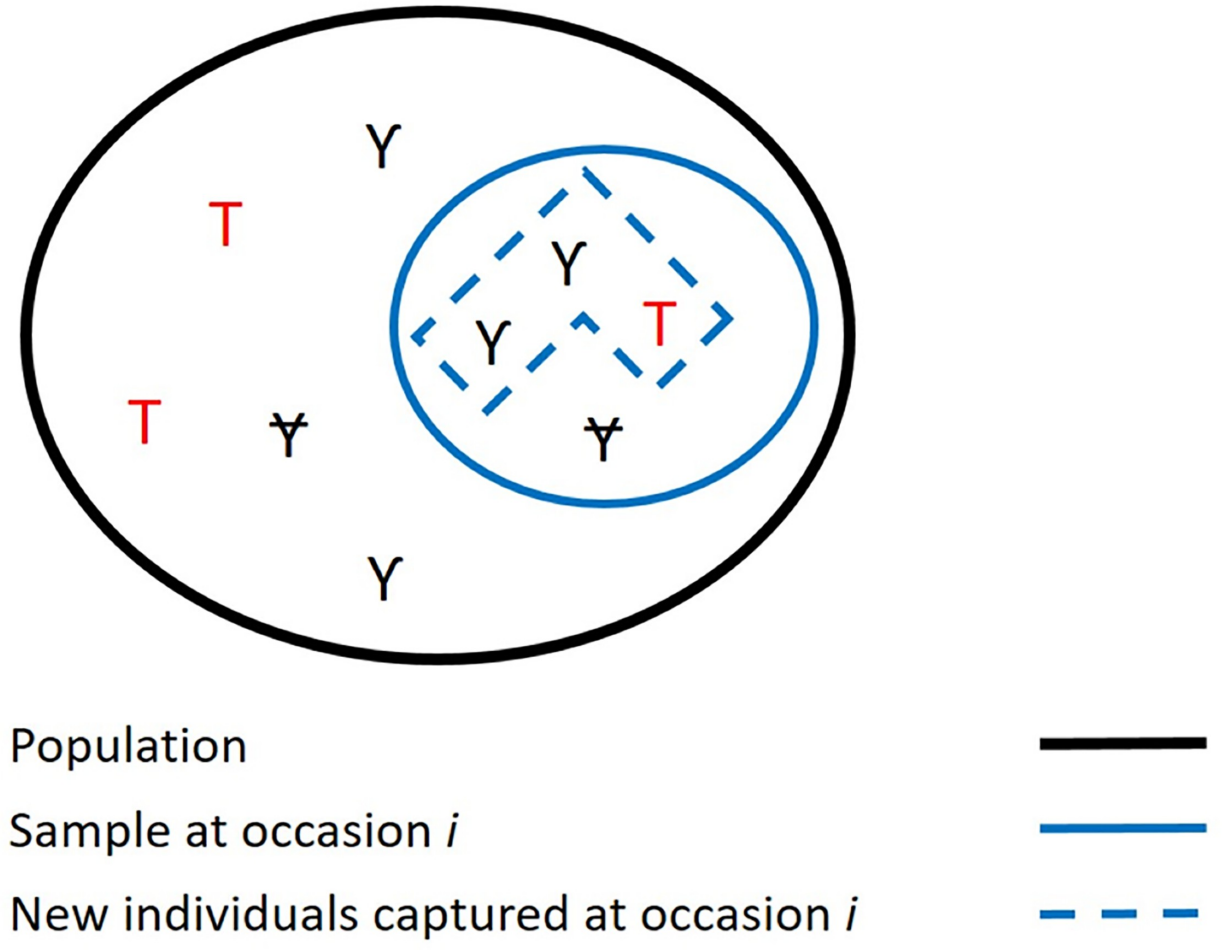
Transients proportion in a sample and a population under a capture-recapture monitoring. Ɏ: Resident marked, Ƴ: Resident unmarked and T: Transient. In this particular example, T: proportions in transients in the population equals 3/10 and τ: proportion of transients among newly captured equals 1/3.

As transients, by definition, cannot be re-observed after their initial encounter, detection of transients is intrinsically zero after the first encounter and this must be enforced in the model. In this parametrization, we may test for environmental or individual factors or variables potentially affecting the transience probability by modeling the initial state probabilities. This may serve for instance to detect whether there are more transients at some occasions or under some environmental or individual conditions. However, one may be cautious to note that the proportion of transients among the newly marked, τ, which is what we estimate as *Tr*, differs in general from the proportion in the whole population, T, the interesting parameter (Fig 1). They differ because of two unrelated features: the relative detectability of transients and residents, and the proportion of the population that is already marked. That τ differs from T is not by itself a problem when one wants to assess the influence of a variety of covariates as long as the relationship is monotonous. Thus, if one may assume that the relative detectability of transients and residents and that the proportion of the population that is already marked do not change, τ is a valid proxy of T. The second point is yet probably untrue during the early years of any study when the proportion marked tends to increase. Pradel et al. [9] have derived a formula relating τ and T, when T, ϕ, and *p* are constant. This may serve as a basis to decide for how many of the early occasions τ is not a valid proxy of T. [9] also provide the exact formula relating τ and T at each occasion when detectability can be assumed the same for residents and transients; the correction factor is then simply the expected proportion of unmarked in the sample. If samples are large enough, the observed proportions can be used instead and an estimation of T can be implemented. Alternatively, an independent scan of the population can be used to estimate this proportion.

#### Transience as a transition (C)

In this parametrization, the individual states considered are: Individual alive (A) and dead (D), this last state not being observable. The possible events are: not seen (0) and seen (1). In this case, the transition part of the model involves two steps. The first one accounts for the transience probability, i.e. the probability that an individual following its first encounter dies or departs definitively from the study area as a direct or indirect consequence of the initial observation (see ‘capture effect’ and ‘cost of first reproduction’ above), and the second one accounting for the survival process of those that remain.

Initial State:

$$\begin{array}{cc} A & D\\ 1 & 0 \end{array}$$

Transition matrices:

step 1: Transience

AD

$$Tr=\begin{array}{c} A\\ D \end{array}\left(\begin{array}{cc} 1-Tr & Tr\\ 0 & 1 \end{array}\right)$$

step 2: Survival

AD

$$S=\begin{array}{c} A\\ D \end{array}\left(\begin{array}{cc} \phi & 1-\phi \\ 0 & 1 \end{array}\right)$$

Event probabilities matrix:

01

$$E=\begin{array}{c} A\\ D \end{array}\left(\begin{array}{cc} 1-p & p\\ 1 & 0 \end{array}\right)$$

In this parametrization, we estimate the transience probability at the first step of transitions. In this step, we may test for variables driving transience probability. As the transience effect is specific to the first encounter, the probability of transience at later occasions is equal to zero.

### Which parametrization should I use?

**Fig 2:**
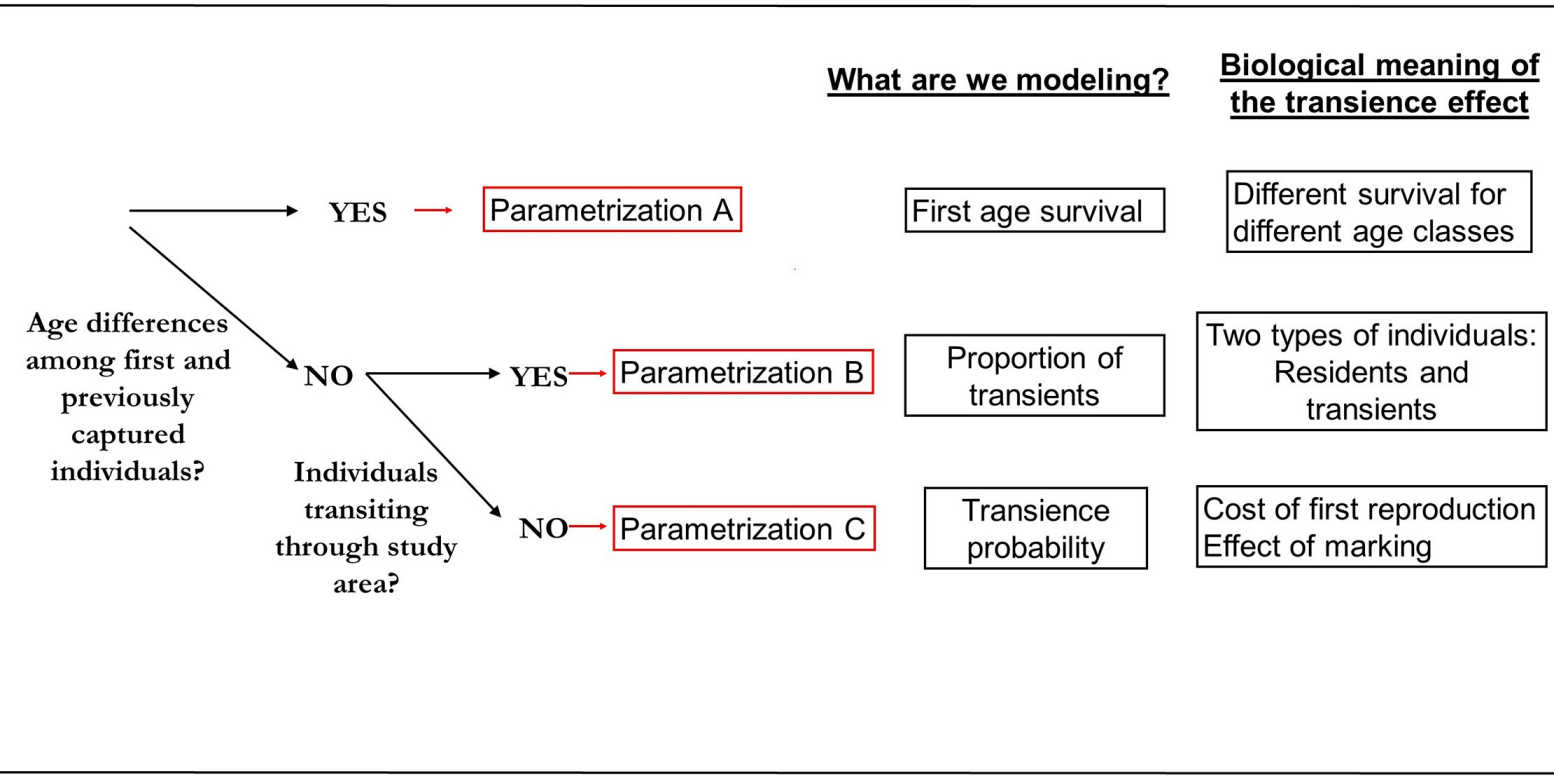
Decision tree for selecting the best parametrization to include a transience effect.

To choose the best parametrization to analyze our data we should consider: a) are we interested in these individuals that generate this transience effect? b) do we need to assess factors affecting the transience probability? and when possible c) which is the biological origin of our detected transience effect. Based on that, we are going to choose the most appropriate capture-recapture parametrization (Fig 2). If all individuals are marked at the same age, and there are consequential differences due to age between individuals marked and individuals captured simultaneously but previously observed, parametrization A will be useful. If we rather suspect that some individuals that have been sampled are transiting through our study area and do not belong to our study population (e.g. studies at stopover sites or at breeding sites but not sampling sure breeders), we suggest to choose parametrization B. In case we suspect that some individuals may be affected by the first sampling procedure (e.g. effect of marking) or by other events occurring during their first sampling (e.g. effect of first breeding attempt), we suggest to use parametrization C. If we do not know a priori the biological causes of the transience effect, we suggest choosing parametrization C, because it is the one that would involve the fewest biological assumptions and the most flexible for hypotheses testing.

Decision tree for selecting the best parametrization to render a transience effect in capture-recapture models. Parametrization types are A: Transience effect as an age effect, B: Transience effect as an individual state and C: Transience effect as a transition.

### Worked example

As an example, we analyze a long-term data set (1999–2018) of adult Scopoli’s shearwaters *Calonectris diomedea diomedea* breeding at the Aire islet (Menorca, western Mediterranean) (see S1 File on supporting information for details on the study species and study area). We detected a transience effect with the GOF test ($\chi_{17}^{2}$ = 66.42, p<0.001). Given that our database only include adults, first release does not correspond to a specific age, thus parametrization A is not the best option to model the transience effect. Additionally, as we sample individuals breeding at the nest, all our individuals are surely residents. According to Fig 2, parametrization C is the best analytical procedure here. For the sake of illustration and comparison, we are nonetheless going to present the other two parametrizations.

Under each analytical procedure and based on previous results [28], we build different models to test for constancy, time dependency, and to test for an environmental covariate potentially affecting transience and survival probabilities, namely the Southern Oscillation Index (SOI) (http://www.cru.uea.ac.uk/cru/data/soi/soi.dat) (see S1 File on supporting information for the practical implementation of the different multi-event modeling approaches). Model selection relies on QAICc (i.e., the Akaike Information Criterion duly corrected for small sample size) [29].

Results from our case study evidence some differences between parametrizations (Tables 1 and 2). Parametrizations B and C are identical from a statistical point of view and give us exactly the same estimates (Tables 1 and 2); they only differ from a conceptual point of view. However, parametrization A estimates a different parameter and, when including covariates, the models differ also from a statistical point of view (Tables 1 and 2). If adult survival estimates obtained from each parametrization are identical, parametrization A gives survival of individuals captured for the first time and parametrization B and C give us the transience probability, i.e. the probability that a newly caught individual disappear from the population (die or permanently emigrate) beyond what is expected based on the behavior of the previously caught individuals from the same sample. Thus caution should be made when choosing between each analytical procedures, especially between parametrization A versus B or C.

**Table 1:** Model selection and hypothesis tested.

Model	Param.	Transience	Survival	Recapture	np	Deviance	QAICc	Delta QAICc	*w*_*i*_
**1**	**B**	**SOI**	**ctant**	**t**	**22**	**1970,57**	**2015,82**	**0,00**	**0,21**
**2**	**C**	**SOI**	**ctant**	**t**	**22**	**1970,57**	**2015,82**	**0,00**	**0,21**
**3**	**A**	**-**	**a1(SOI),a2**	**t**	**22**	**1970,86**	**2016,11**	**0,29**	**0,18**
**4**	**A**	**t**	**ctant**	**t**	**39**	**1934,44**	**2016,37**	**0,55**	**0,16**
**5**	**C**	**t**	**ctant**	**t**	**39**	**1935,14**	**2017,06**	**1,25**	**0,11**
**6**	**B**	**t**	**ctant**	**t**	**39**	**1935,14**	**2017,06**	**1,25**	**0,11**
7	B	ctant	SOI	t	22	1981,24	2026,49	10,67	0,00
8	C	ctant	SOI	t	22	1981,24	2026,49	10,67	0,00
9	C	ctant	ctant	t	21	1983,65	2026,79	10,97	0,00
10	B	ctant	ctant	t	21	1983,65	2026,79	10,97	0,00
11	A	-	a1,a2	t	21	1983,65	2026,79	10,97	0,00
12	A	-	a1,a2(SOI)	t	22	1983,64	2028,88	13,07	0,00
13	B	ctant	ctant	ctant	3	2089,70	2095,72	79,90	0,00
14	A	-	a1,a2	ctant	3	2089,70	2095,72	79, 90	0,00
15	C	ctant	ctant	ctant	3	2089,70	2095,72	79, 90	0,00

Model selection and hypothesis tested with Multievent modelling. Param. = Parametrization type: A:Transience as an age effect, B: Transience as an individual state and C: Transience as a transition; np = number of parameters; *w*_*i*_ = weight of model *i*. a1 = individuals seen for the first time; SOI = Southern Oscillation index as a covariate; t = time varying; ctant = constant over time. QAICc: Akaike information criterion corrected for small sample size; DeltaQAICc: the QAICc difference between the current model and the one with the lowest QAIC value; ***w***_***i***_: Akaike’s weight of the model. The models best fitting our data are shown in bold.

**Table 2:** Mean *transience* and adult survival probabilities.

	A	B	C
	*transience* as an age effect	*transience* as an individual state effect	*transience* as a transition
Transience^1^	0.66 (0.57–0.73)	0.28 (0.19–0.38)	0.28 (0.19–0.38)
Adult survival	0.90 (0.88–0.92)	0.90 (0.88–0.92)	0.90 (0.88–0.92)

Estimates of mean *transience* and adult survival probabilities (and 95% Confidence Intervals in parentheses) for Scopoli’s shearwaters on the colony of Aire (from models 9, 10 and 11, Table 1).

^*1*^*Transience* in parametrization A is not the transience probability, but the survival probability of all individuals captured for the first time.

Previous studies have rejected an effect of marking in this Scopoli’s shearwater study with an even more invasive type of marking [30]. Hence, we interpret the transience effect in this dataset as a non-negligible cost of first reproduction.

## Discussion

We provide here an updated framework for including a transience effect in capture-recapture models. Even if the implementation is detailed for working in the Multievent framework, the same conceptual ideas can be implemented as state space models.

We advocate for treating transience as an age effect (parametrization A) only in those cases where there is evidence that the transient effect is due to a difference in age of individuals captured for the first time. Otherwise, and especially in those cases where we want to infer factors driving the transience probability, we strongly encourage the use of parametrizations B and C, treating transience as an individual state or as a transition. Parametrizations B and C are identical from a statistical point of view and will give us exactly the same estimates. However, we suggest the use of parametrization B if we suspect the existence of “transients and residents” in our study sample, and parametrization C on the other scenarios.

Based on a good knowledge of natural history features of the study system and the biological model used, the biological reasons of the transience effect should be the guide for choosing the appropriate analytical procedure. In our case example we had enough previous information to determine the most plausible biological meaning of the transience effect, however, which is the best choice when the demography of the focal species is poorly known or when multiple causes (e.g., natal dispersal, cost of first reproduction, age-dependent survival) may lead to a transience effect? As suggested, the age of marking should guide us to the first decision; if we do not know the age at marking, or if individuals are marked both as juveniles and adults, we should avoid parametrization A. Then between parametrizations B and C, if we do not have enough information to guess the most plausible biological meaning of the transience effect, we suggest to choose parametrization C, as previously mentioned, and we could attempt to discriminate between biological meanings of the transience effect. For example, we could use a measure of the intensity of manipulation at each occasion or a change in the marking technique at some point for assessing a marking effect; or the comparison of luring or sampling methods to detect the presence of true transients.

This framework will help clarifying the ideas and terminology when dealing with a transience effect. Additionally, these parametrizations directly estimate the “transience probability” and allow testing biological hypotheses about factors driving this probability; this open new and powerful ways for the detection and exploration of factors affecting ecological processes such as the costs of the first reproduction or the deleterious effects of some types of marking in natural populations.

## Supporting information

S1 File Practical implementation and specification of the different multi-event modeling approaches in program E-SURGE. (PDF)
