# Supplementary material for: Transience effect in capture-recapture studies: The importance of its biological meaning
Source: PLoS One. 2019 Sep 19;14(9):e0222241. doi: 10.1371/journal.pone.0222241 (PMC6752852; doi:10.1371/journal.pone.0222241)
Supplement: S1 File — (PDF) [file pone.0222241.s001.pdf]

Appendix- Supporting Information

Transience effect in capture-recapture studies: the importance of its biological meaning

Meritxell Genovart and Roger Pradel

Practical implementation and specification of the different multi-event modeling approaches in program E-SURGE

We show here a practical implementation with program E-SURGE of three different parametrizations that could be used for including a transience effect in a Multievent capture-recapture analysis. As explained in the main document, transience effect can be approached as an age effect (A), as an individual state (B) and as a transition (C). We use a data set of adult Scopolis shearwater *Calonectris diomedea diomedea* breeding at Illa de l'aire colony (Menorca) from 1999 to 2018. We compare results obtained with each parametrization and show how to test for covariate effects on the transience probability.

Study Species and study area

Scopoli's shearwater *Calonectris diomedea diomedea* is a long-lived Procellariiform which carries out long-distance migration, with most individuals arriving in the Mediterranean by early March and breeding from May to October (Cramp & Simmons, 1977; de los Reyes-González & González-Solís, 2016). The species lays one egg and both members of the pair share in incubation and chick-feeding (Cramp & Simmons, 1977; del Hoyo et al., 1992; Sánchez-Codoñer & Castilla, 1997).

Data were collected from a rat- and carnivore-free western Mediterranean colony, the Aire Island in Menorca (39°48′ N, 4°17′E), in the Balearic Archipelago, holding ca. 60 breeding

pairs. At this colony, adults and chicks are trapped during daylight on their nesting burrows and marked with stainless-steel bands with a unique code to allow identification, or recaptured if they had been previously marked.

Multi-event modeling

For our analyses, we only considered individuals captured as adults. All our models have been developed in the multi-event capture-recapture framework and models were fitted in the program E-SURGE (Pradel, 2005; Choquet *et al.*, 2009b).

Based on previous results (Genovart *et al.*, 2013), and to illustrate how to test the effect of covariates with these parametrizations, we built different models to test for a constant and time variant survival, and for an effect of the Southern Oscillation Index (SOI) (http://www.cru.uea.ac.uk/cru/data/soi/soi.dat) on both transience and survival probabilities. Our capture-recapture dataset includes data from 1999 to 2018. Model selection relied on QAICc (i.e., the Akaike Information Criterion duly corrected for small sample size) (Burnham & Anderson, 2002).

We detail below the three different analytical ways to include a transience effect in our capture-recapture models.

A) TRANSIENT AS AN AGE EFFECT

In this parametrization transience would correspond to those individuals of age 1 that have different survival or permanent dispersal than those older individuals.

Thus, our syntaxes for modelling survival should include at least two age classes. We could test for covariates affecting the survival of different age classes. Here we do not directly test the effect of the covariate on the transience probability but on the first age survival.

The individual states considered are:

A: Alive

D: Dead

The possible events are:

0: not observed

1: Seen alive breeding

The symbols for parameters are:

ϕ**:** Survival probability

p: Recapture probability

<u>Initial State probabilities</u>

**A**	**D#**
*	-

This last column is hidden by default in E-Surge

<u>Transition probabilities,</u>

Survival

	**A**	**D**
**A**	ϕ	*
**D**	-	*

Event probabilities: observation process.

step 1: Recapture

	**0**	**1**
**A**	*	p
**D**	*	-

B) TRANSIENT AS A STATE

With this parametrization we consider to be transient as a possible individual state and estimate the probability to be transient at the initial state matrix. Modeling initial state probabilities, we can assess which factors or covariables affect the transience probability.

The individual states considered are:

AT: Alive and transient

AR: Alive and resident

D: Dead

The possible events are:

0: Not observed

1: Seen alive breeding

The symbols for parameters are:

Tr : Transience probability

ϕ**:** Survival probability

p: Recapture probability

In E-SURGE, the pattern matrices are:

<u>Initial State probabilities</u>

**AT**	**AR**	**D**[#]
Tr	*	-

[#] This last column is hidden by default in E-Surge

<u>Transition probabilities,</u>

step 1: Survival

	**AT**	**AR**	**D**
**AT**	**-**	**-**	*
**AR**	-	ϕ	*

D	-	-	*

Event probabilities: observation process.

step 1: Recapture

	**0**	**1**
**AT**	*	p
**AR**	*	p
**D**	*	-

C) TRANSIENT AS A TRANSITION

With this parametrization we assume that transience is a possible step at the transition matrices. We thus model transient probability at the first step of transition. Transience probability at ages older than 1 should be fixed to zero.

The individual states considered are:

A: Alive

D: Dead

The possible events are:

0: Not observed

1: Seen alive breeding

The symbols for parameters are:

Tr : Transience probability

ϕ**:** Survival probability

p: Recapture probability

<u>Initial State probabilities</u>

A	D#
*	-

This last column is hidden by default in E-Surge

<u>Transition probabilities,</u>

step 1: Transience

	A	D
**A**	*	*Tr*
**D**	-	*

step 2: Survival

	A	D

**A**	ϕ	*
**D**	-	*

Event probabilities: observation process.

step 1: Recapture

	**0**	**1**
**A**	*	p
**D**	*	-

Detailed example for fitting models 1 (parametrization B), 2 (parametrization C) and 3 (parametrization A) with program E-SURGE

Model 1 (parametrization B)

In this parametrization the number of states is changed to 3. Then we go through GEPAT to specify the patterns as indicated above.

GEPAT

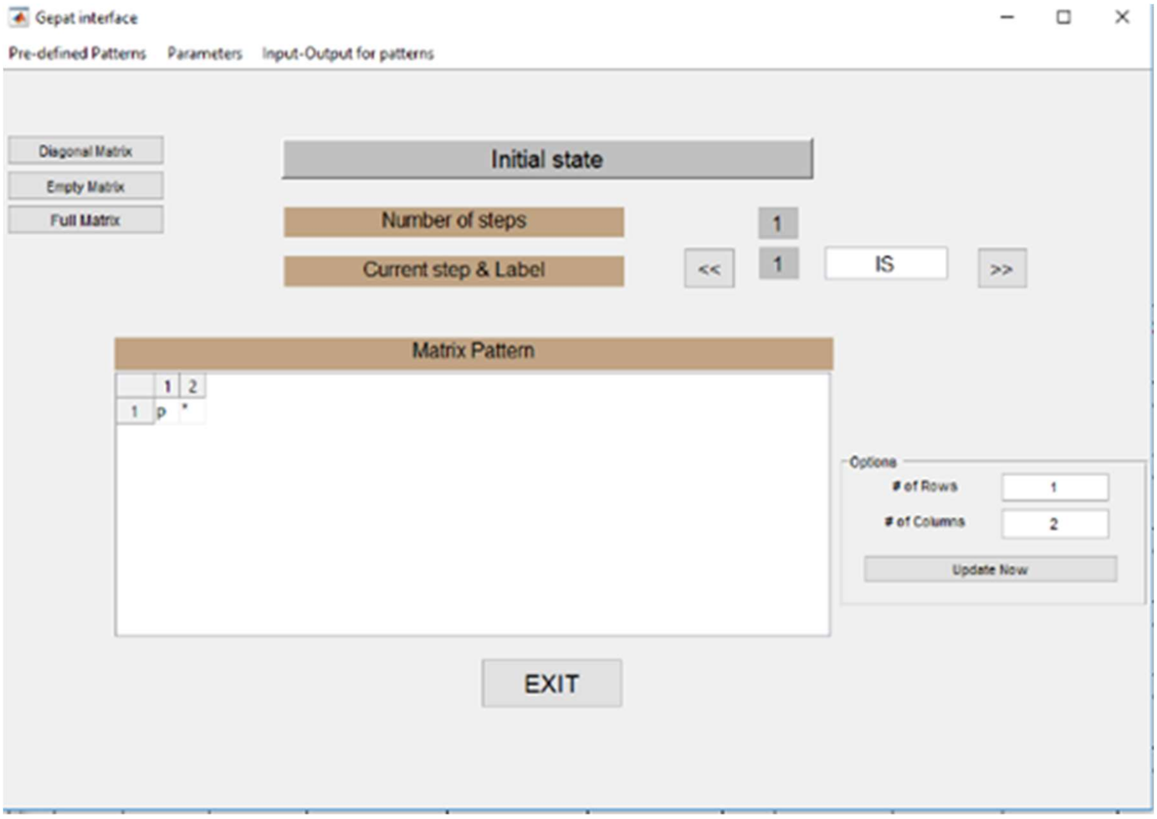
Gepat interface
Pre-defined Patterns
Parameters
Input-Output for patterns
Diagonal Matrix
Empty Matrix
Full Matrix
Initial state
Number of steps
1
Current step & Label
<<
1
IS
>>
Matrix Pattern
Options
# of Rows
1
# of Columns
2
Update Now
EXIT

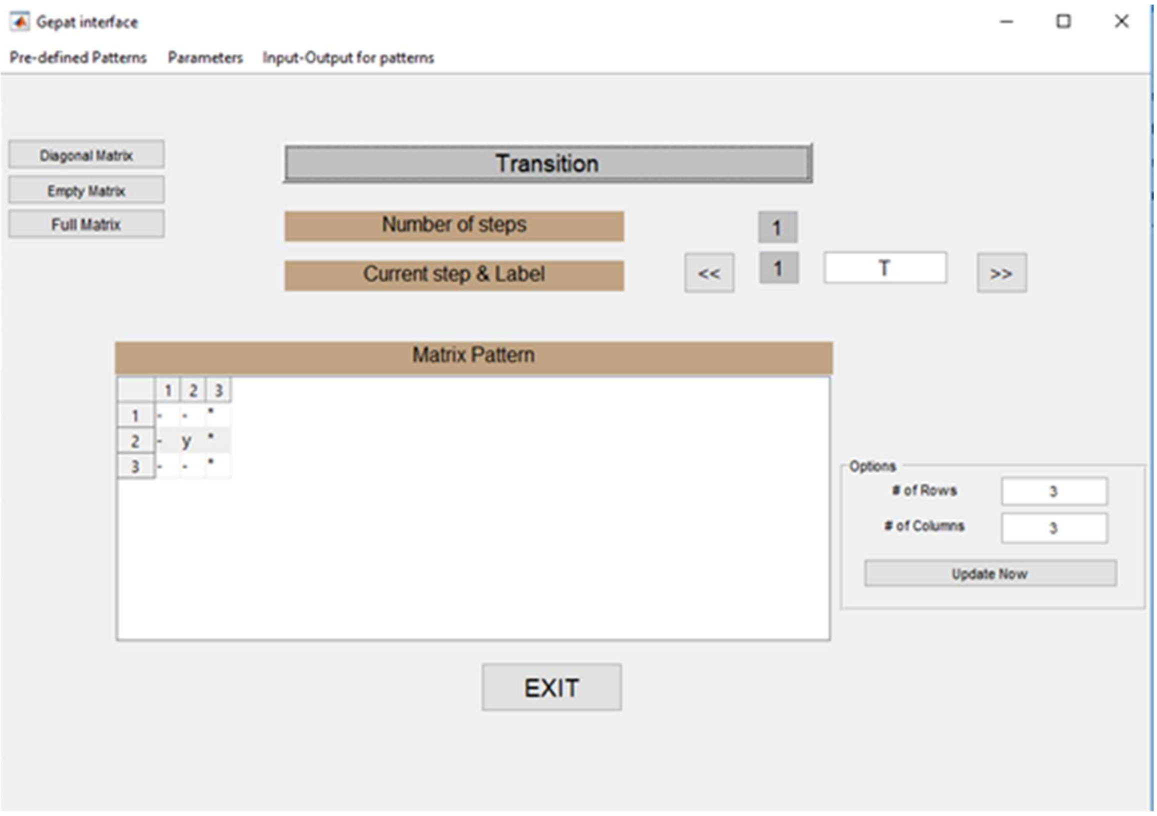
Gepat interface
Pre-defined Patterns
Parameters
Input-Output for patterns
Diagonal Matrix
Empty Matrix
Full Matrix
Transition
Number of steps
1
Current step & Label
<<
1
T
>>
Matrix Pattern
Options
# of Rows
3
# of Columns
3
Update Now
EXIT

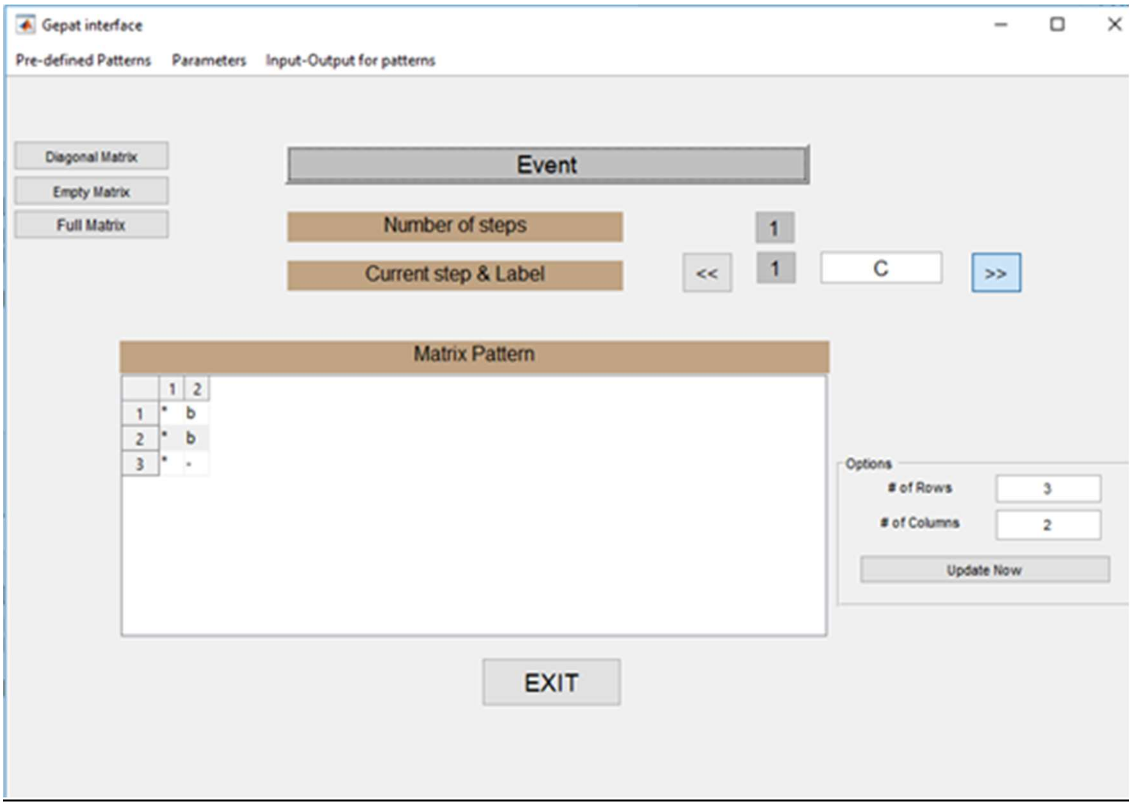

GEMACO

In this parametrization the probability of being a transient is modeled at the initial state. In this model the annual probability of being a transient depend on the Southern Oscillation index.

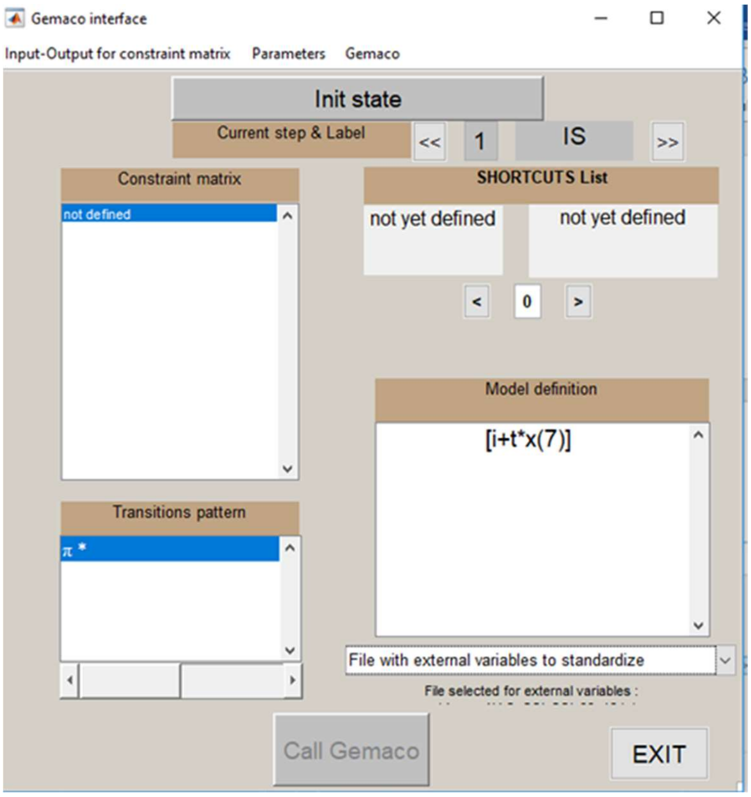

In this model survival probability is assumed to be constant over the study period.

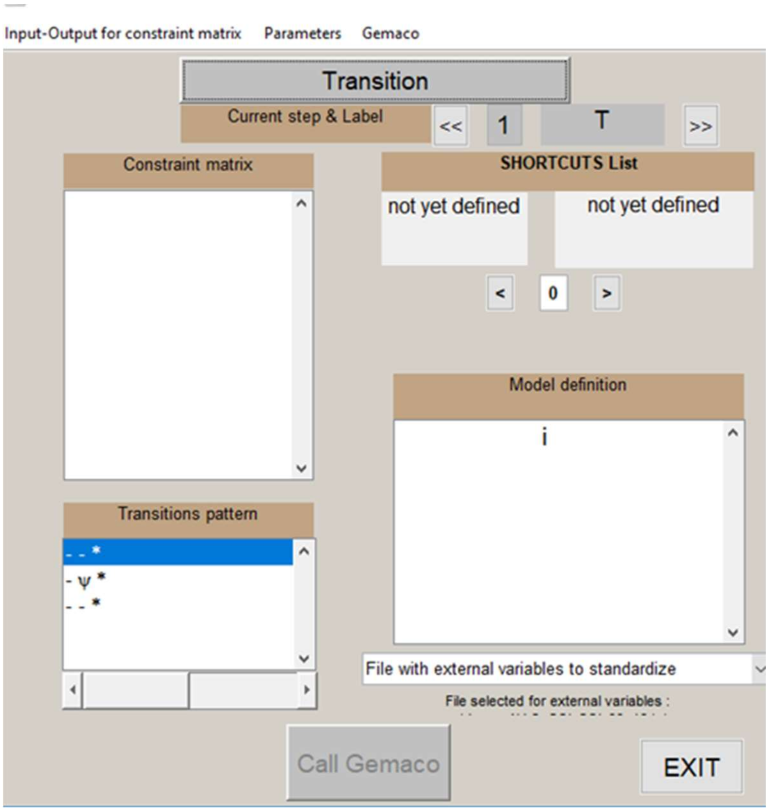

As the effort of sampling at colonies is variable over the study period, all the models assume the recapture probability is time variant.

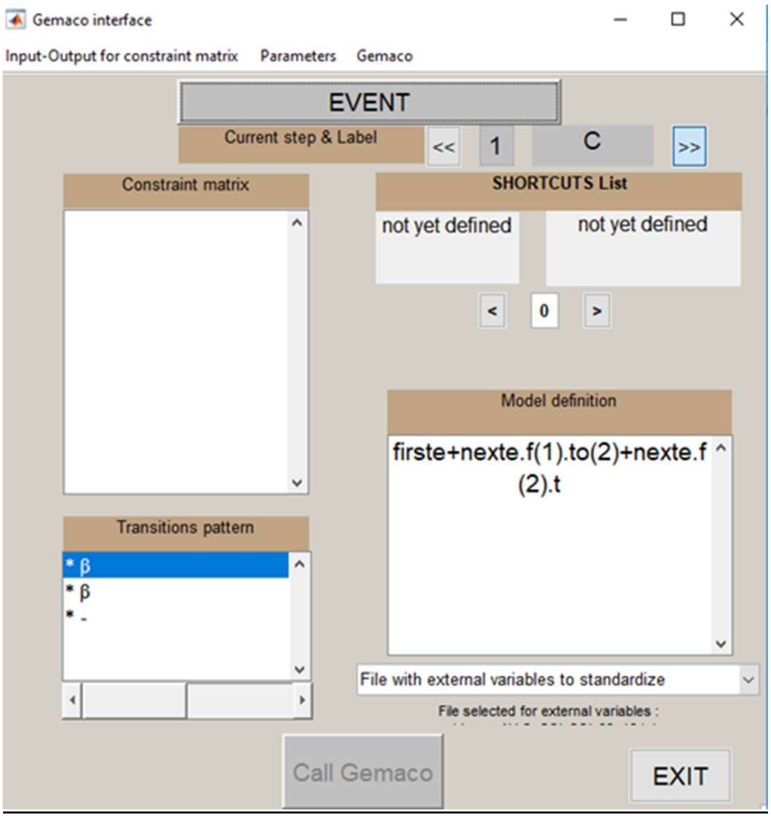

Under this scenario we should fix as usual the probability of the first capture to one (firste =1), but we also should fix the probability of being observed as a transient to zero after the first capture (nexte.f(1).to(2) =0).

Model 2 (parametrization C)

In this parametrization the number of states is left to 2. Then we go through GEPAT to specify the patterns as indicated above.

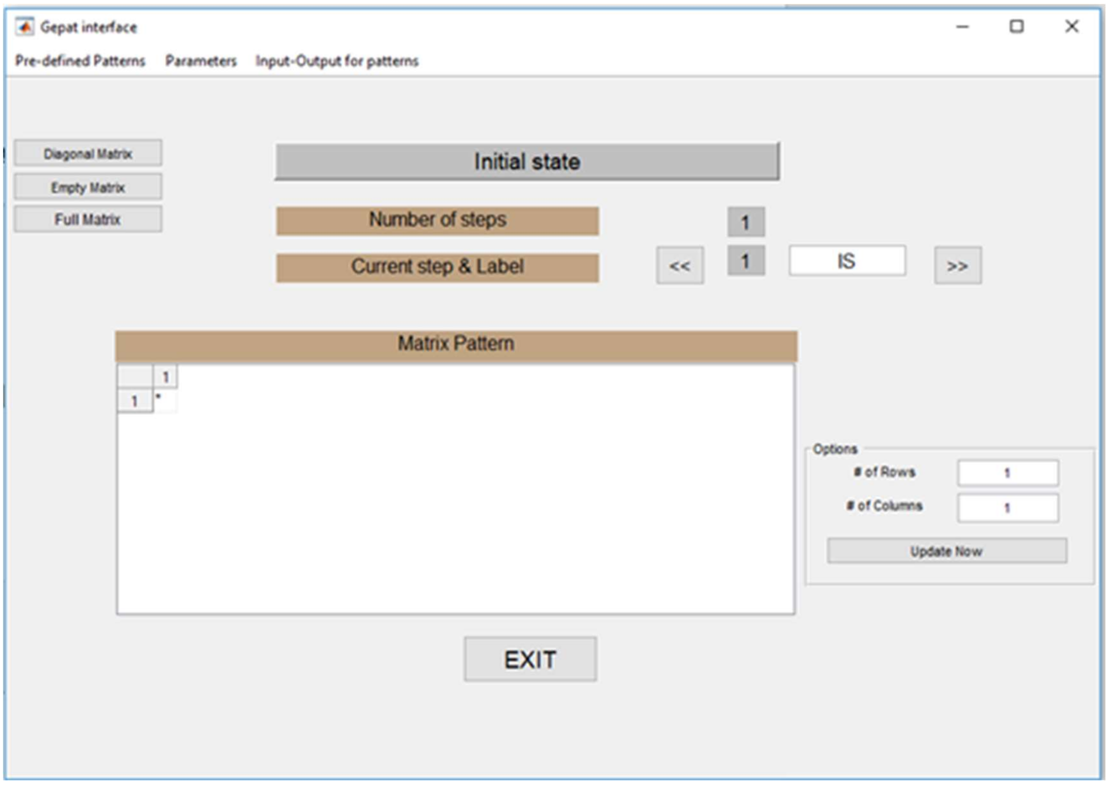

In this parametrization Transition is divided in two steps, one for the transience probability and the second one for survival.

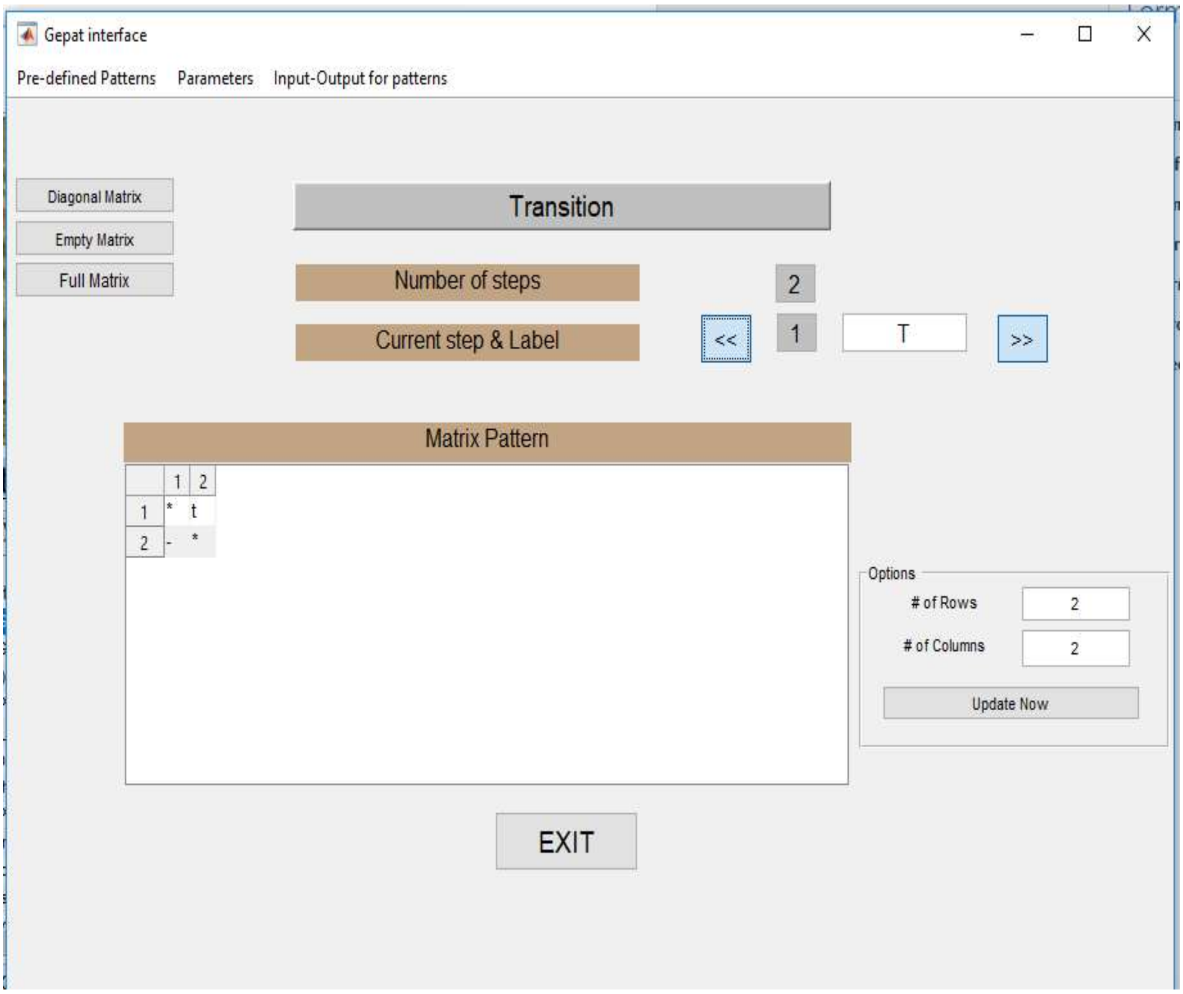
Gepat interface
Pre-defined Patterns
Parameters
Input-Output for patterns
Diagonal Matrix
Empty Matrix
Full Matrix
Transition
Number of steps
2
Current step & Label
<<
1
T
>>
Matrix Pattern
1 2
1 * t
2 - *
Options
# of Rows 2
# of Columns 2
Update Now
EXIT

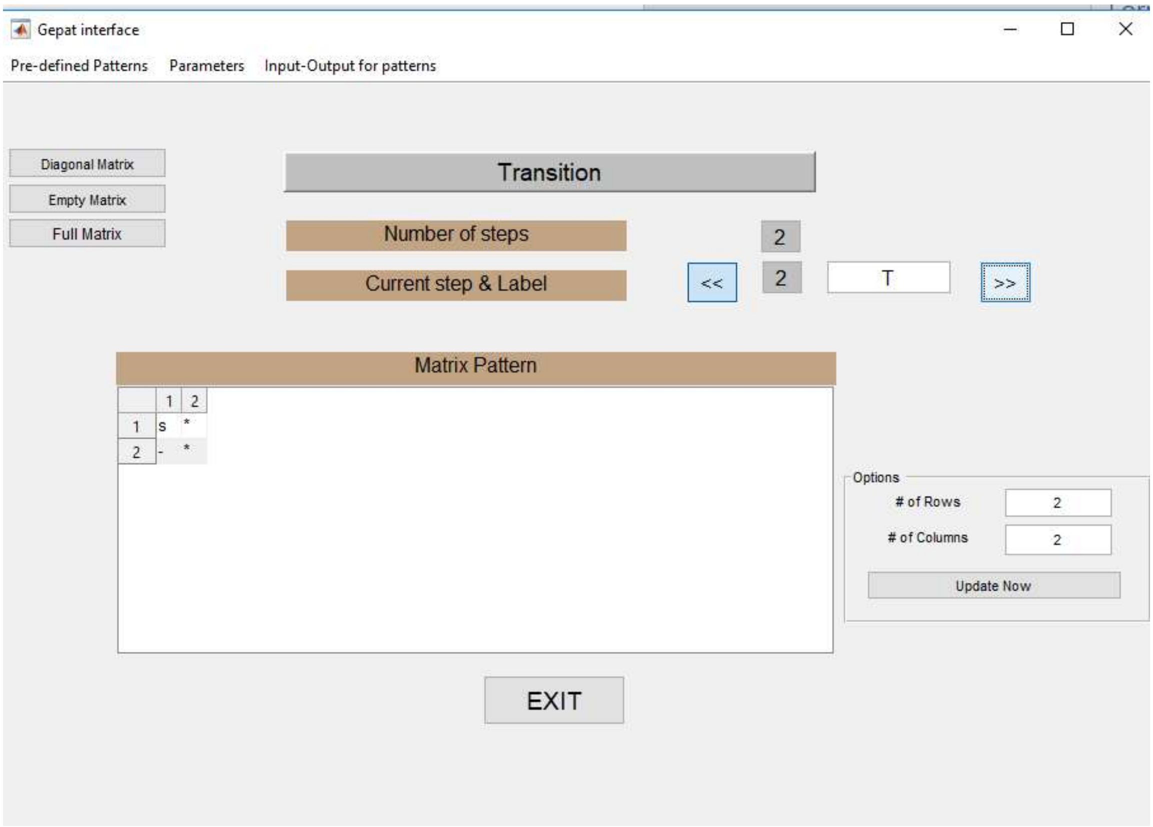
Gepat interface
Pre-defined Patterns
Parameters
Input-Output for patterns
Diagonal Matrix
Empty Matrix
Full Matrix
Transition
Number of steps
2
Current step & Label
<<
2
T
>>
Matrix Pattern
1 2
1 s *
2 - *
Options
# of Rows
2
# of Columns
2
Update Now
EXIT

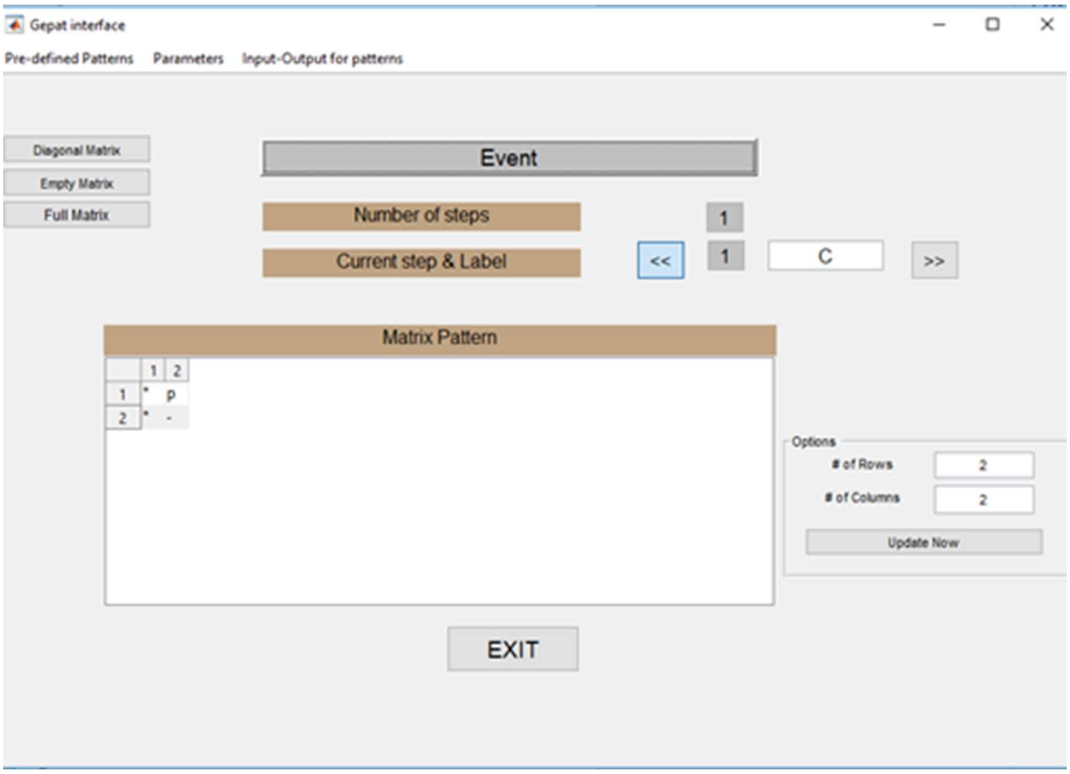
Gepat interface
Pre-defined Patterns
Parameters
Input-Output for patterns
Diagonal Matrix
Empty Matrix
Full Matrix
Event
Number of steps
1
Current step & Label
<<
1
C
>>
Matrix Pattern
Options
# of Rows
2
# of Columns
2
Update Now
EXIT

GEMACO

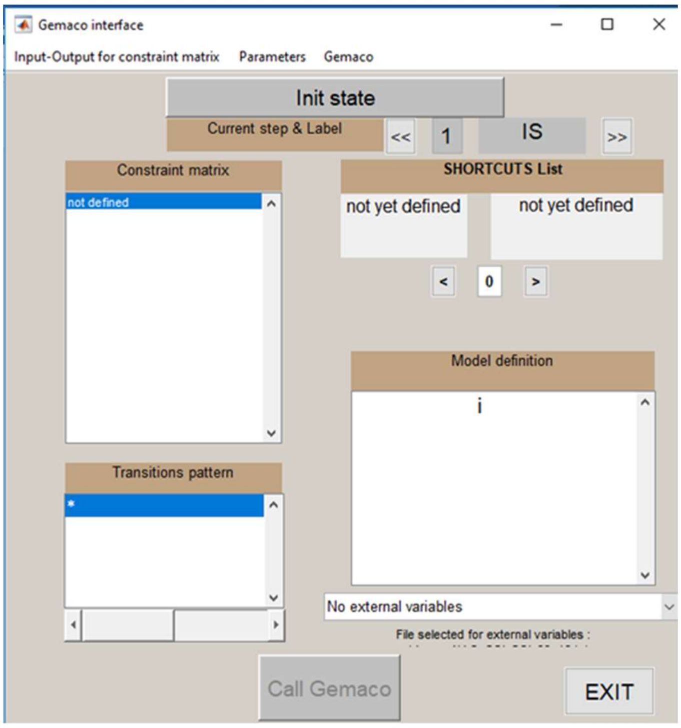
Gemaco interface
Input-Output for constraint matrix
Parameters
Gemaco
Init state
Current step & Label
<<
1
IS
>>
Constraint matrix
not defined
SHORTCUTS List
not yet defined
not yet defined
<
0
>
Model definition
Transitions pattern
No external variables
File selected for external variables :
Call Gemaco
EXIT

In this model, transience probability is assumed to depend on the Southern Oscillation index.

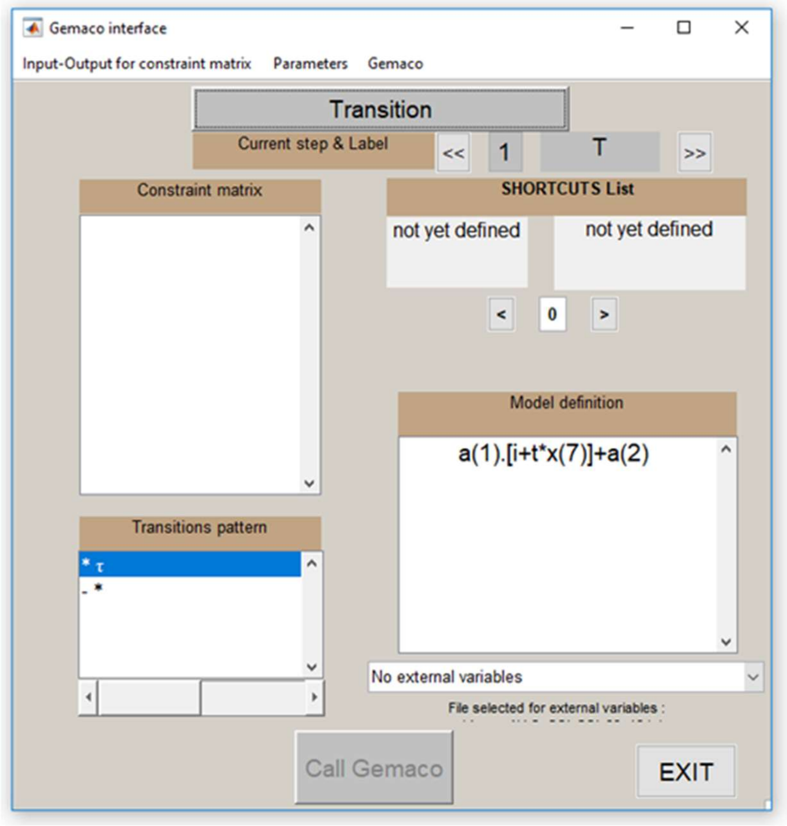

And survival probability is assumed constant over the study period.

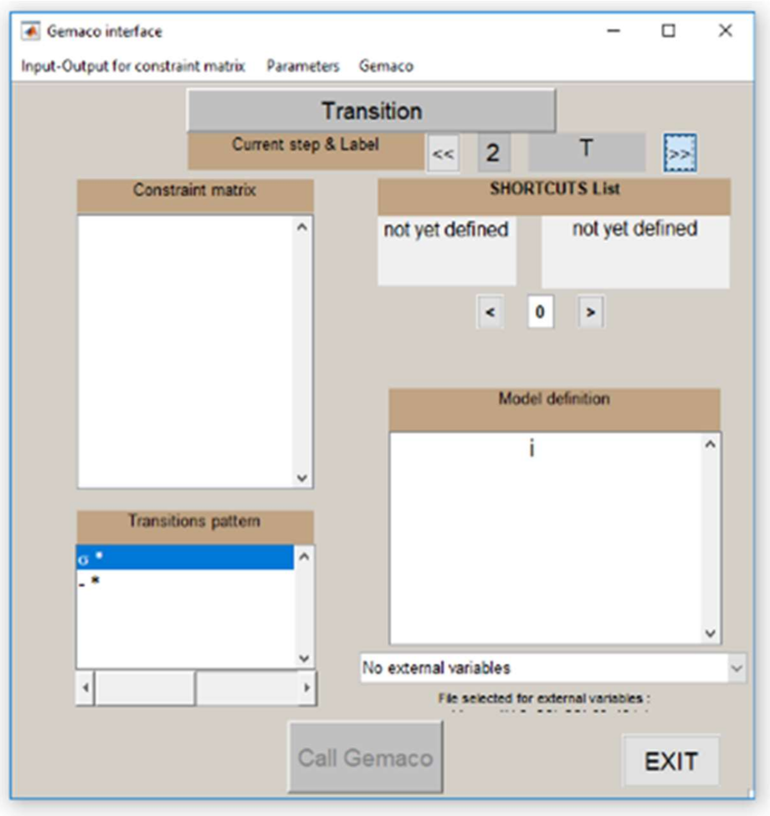

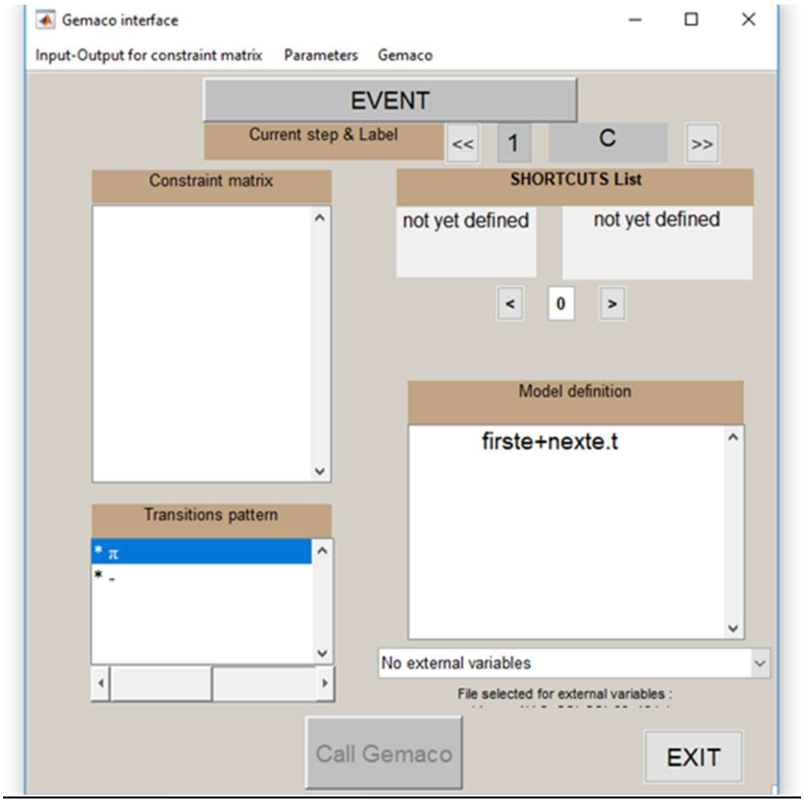

As usual, under this scenario we should fix as usual the probability of the first capture to one (firste =1) and the transience probability to zero for those individuals older than 1.

Model 3 (parametrization A)

In this parametrization the number of states is left to 2. Then we go through GEPAT to specify the patterns as indicated above.

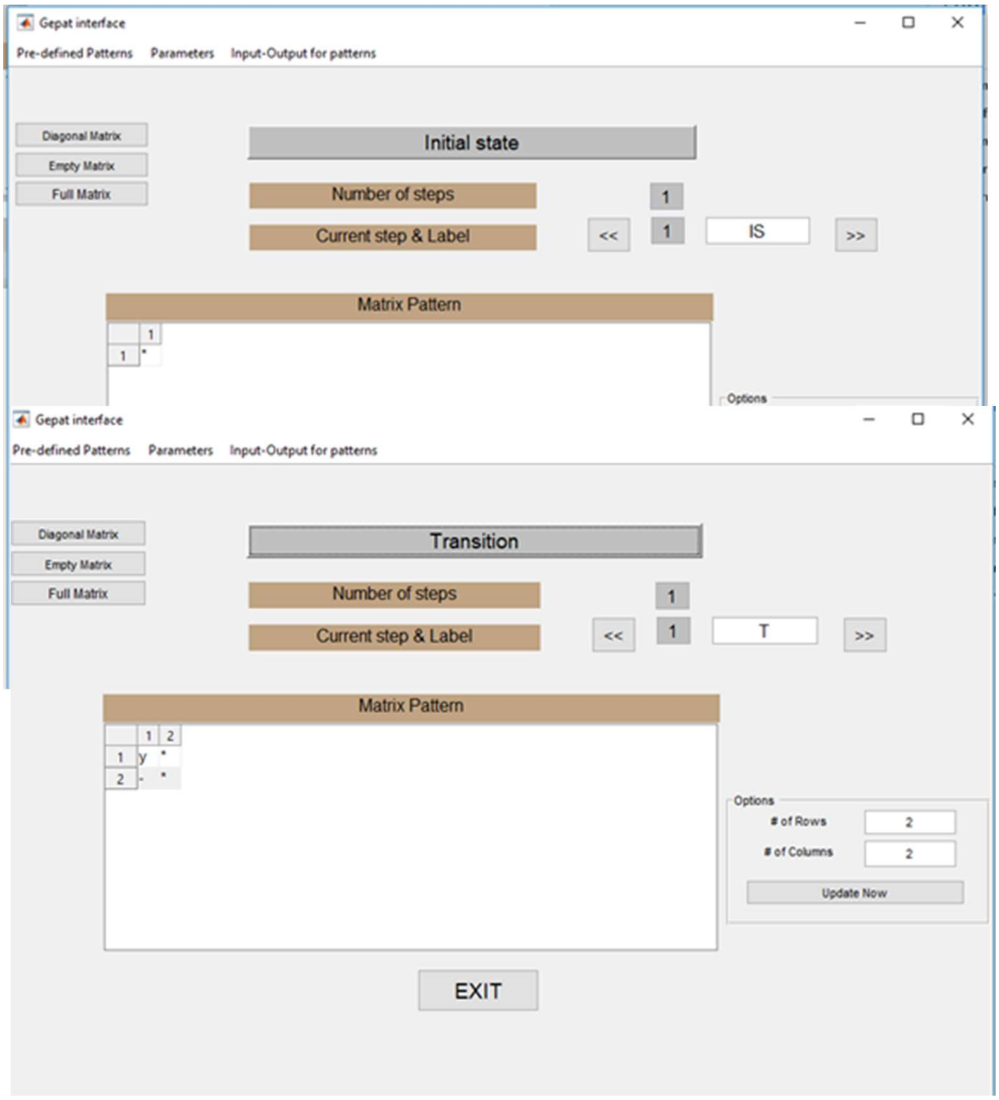

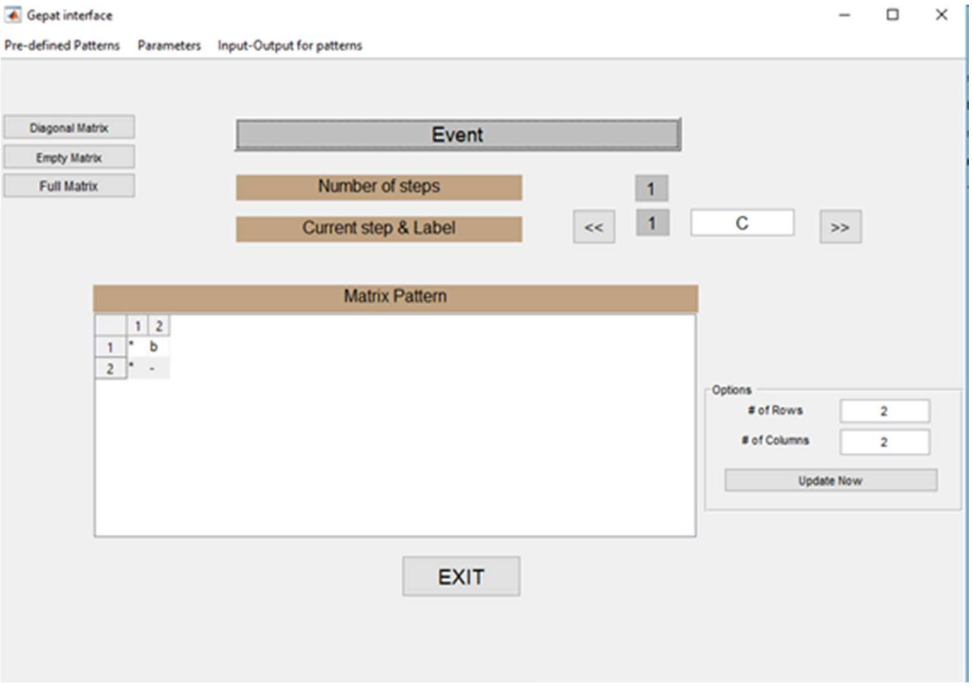
Gepat interface
Pre-defined Patterns
Parameters
Input-Output for patterns
Diagonal Matrix
Empty Matrix
Full Matrix
Event
Number of steps
1
Current step & Label
<<
1
C
>>
Matrix Pattern
Options
# of Rows
2
# of Columns
2
Update Now
EXIT

GEMACO

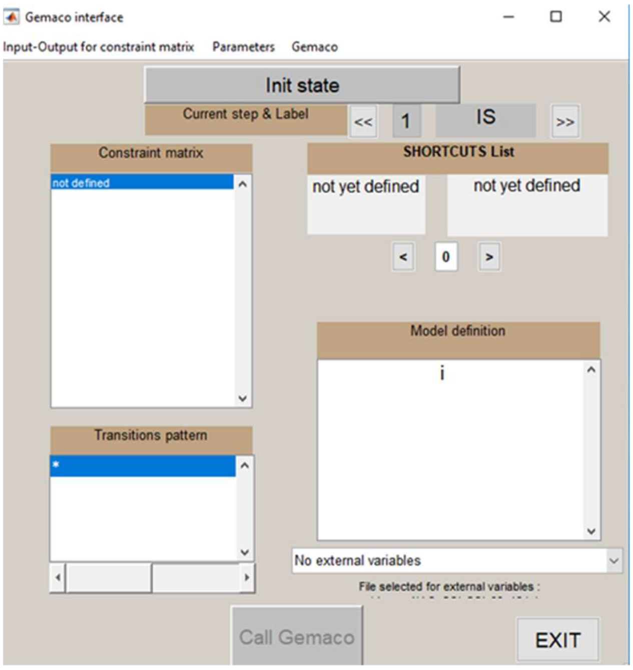
Gemaco interface
Input-Output for constraint matrix
Parameters
Gemaco
Init state
Current step & Label
<<
1
IS
>>
Constraint matrix
not defined
SHORTCUTS List
not yet defined
not yet defined
<
0
>
Model definition
Transitions pattern
No external variables
File selected for external variables :
Call Gemaco
EXIT

In this model, survival probability is assumed to depend on the Southern Oscillation index for those individuals of age 1 and constant over the study period for individuals of age >1.

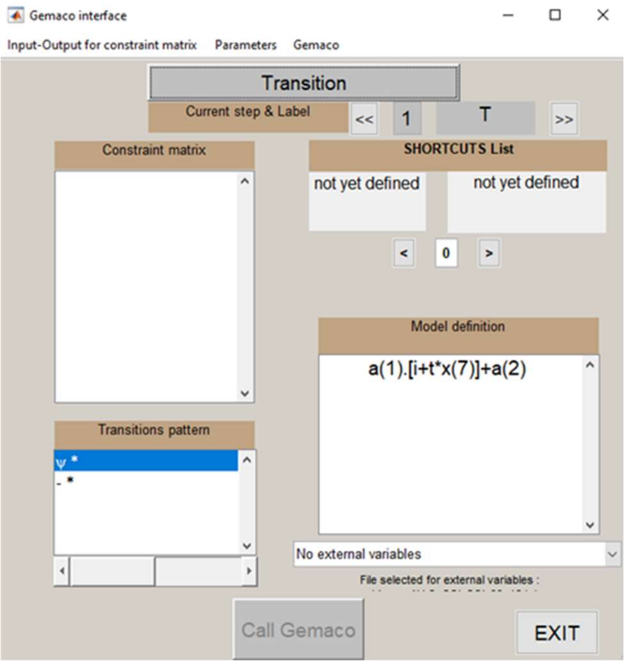

As the effort of sampling at colonies is variable over the study period, all the models assume the recapture probability is time variant.

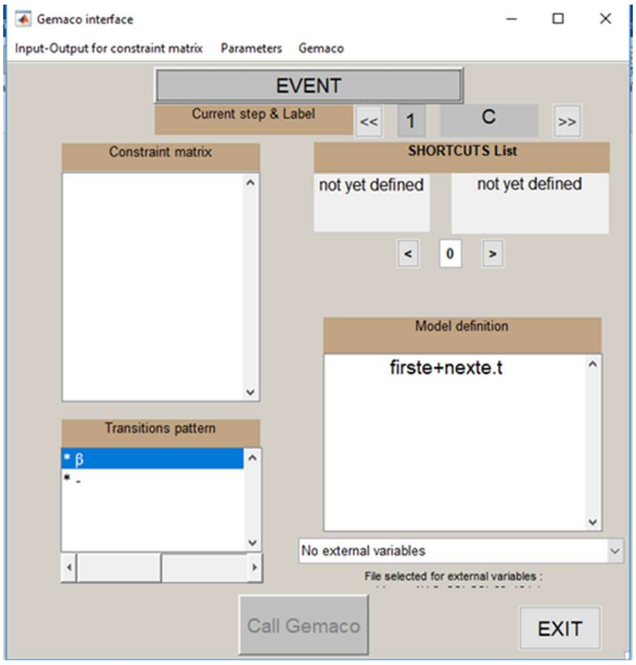

As usual, under this scenario we should fix as usual the probability of the first capture to one (firste =1).
